# Genotype‐Dependent Phenylpropanoid Pathway Specialization in *Prunus avium* Fruits and Leaves Revealed by Untargeted Metabolomics

**DOI:** 10.1111/ppl.71083

**Published:** 2026-08-23

**Authors:** Giuseppe Ardagna, Sofia Gambini, Alessandra Bulgarini, Stefano Negri, Martino Bianconi, Flavia Di Carlo, Stefania Ceoldo, Flavia Guzzo, Mauro Commisso

**Affiliations:** ^1^ Department of Biotechnology University of Verona Verona Italy; ^2^ Department of Biological, Chemical and Pharmaceutical Sciences and Technologies University of Palermo Palermo Italy

**Keywords:** genotype effect, LC–MS, leaf metabolome, phenylpropanoid metabolism, sweet cherry

## Abstract

Phenylpropanoids are specialized metabolites involved in multiple aspects of plant physiology and contribute to fruit quality and human health benefits. In sweet cherry ( *Prunus avium* L.), metabolomic studies have largely focused on fruits, showing that cultivar identity contributes to phenylpropanoid pathway specialization. However, the contribution of genotype to phenylpropanoid organization in other organs, particularly leaves, remains poorly understood. Here, we applied an untargeted metabolomics approach to investigate genotype‐associated phenylpropanoid pathway specialization in sweet cherry leaves, using fruits as a reference organ. Leaves and fruits from seven sweet cherry cultivars (“Early Bigi”, “Burlat”, “Sandra”, “Grace Star”, “Romana”, “Durone Rosso”, and “Ferrovia”), representing early‐, mid‐ and late‐ripening genotypes, were sampled at full fruit maturity across multiple orchards and over two consecutive growing seasons. Distinct organ‐specific phenylpropanoid profiles were observed, with fruits characterized by the accumulation of anthocyanins, flavan‐3‐ols, and simple hydroxycinnamic acids, and leaves enriched in flavonols, hydroxycinnamic acids, and structurally diverse phenylpropanoid derivatives. Multivariate and univariate analyses identified cultivar identity as a major component of phenylpropanoid pathway specialization within each organ. Genotype‐associated differences involved multiple phenylpropanoid subclasses and remained detectable across growing seasons and orchard locations. Overall, these findings indicate differential channeling of the phenylpropanoid pathway between fruits and leaves and show that, within the range of environmental conditions covered by the present experimental design, genotype‐associated variation contributes to shaping phenylpropanoid composition in sweet cherry leaves, revealing a level of organization that had not been previously established in this organ.

## 1 Introduction

*Prunus avium* L. is an arboreal fruit species comprising hundreds of varieties and is of considerable commercial and nutritional importance worldwide (Blando and Oomah 2019; Yang et al. 2025). Sweet cherry fruits are valued not only for the organoleptic qualities but also for the high content of phenolic compounds, which contribute to antioxidant capacity and human health benefits (Kelley et al. 2018; Blando and Oomah 2019; Gonçalves et al. 2019). Fruits are a rich source of bioactive phenolic compounds, including flavonoids, hydroxycinnamic acids, and anthocyanins, which have been widely studied for their antioxidant, anti‐inflammatory, cardioprotective, and neuroprotective properties (Blando and Oomah 2019; Antognoni et al. 2020; Manach et al. 2004; Rana et al. 2022; El‐Saadony et al. 2024; Gonçalves et al. 2024). Clinical and epidemiological studies have shown that cherry consumption may reduce oxidative stress, improve vascular function, and alleviate inflammation‐related conditions such as arthritis and gout (Zhang et al. 2012; Arbizu et al. 2023; Colletti et al. 2025). The growing scientific interest in the health‐promoting properties of sweet cherries has consequently driven numerous efforts to characterize their phytochemical profiles and to understand the factors that influence metabolite accumulation (Commisso et al. 2017; Clodoveo et al. 2023; Mineață et al. 2024; Ceccarelli et al. 2022; Nie et al. 2023).

Among these factors, both genetic and environmental components have been shown to modulate the metabolic composition of sweet cherry fruits, with the genotype emerging as the predominant determinant of both qualitative and quantitative metabolite variation (Commisso et al. 2017; Martini et al. 2017; Boskov et al. 2022). Studies have demonstrated that cultivar identity significantly affects the levels of certain phenolic compounds, including anthocyanins and flavonols, often representing a major source of variation together with growing conditions such as orchard location or climate and the type of rootstock (Commisso et al. 2017; Martini et al. 2017; Boskov et al. 2022).

While much of the current metabolomics research has understandably focused on the fruit, given their economic value and relevance to human health, other tissues such as leaves remain comparatively underexplored, despite their recognized role in the biosynthesis and accumulation of specialized metabolites. In various woody and herbaceous species, leaves have been shown to accumulate substantial levels of phenolic compounds potentially involved in stress response and defense (Andreotti et al. 2006; Kumar et al. 2023; Liu et al. 2024; Jiao et al. 2024; Wang et al. 2025). In sweet cherry, only a limited number of studies have investigated the metabolite composition of leaves, and these have been largely driven by economic considerations, focusing on the recovery of bioactive compounds from leaves regarded as by‐products, while their biological and ecological significance has received comparatively little attention (Dziadek et al. 2019; Nunes et al. 2021). These studies indicate that foliar tissues may contain even higher concentrations of phenolic compounds than fruits, particularly hydroxycinnamic acid derivatives and flavonols.

However, systematic investigations of the sweet cherry leaf metabolome across cultivars and growing seasons remain scarce, and the extent to which leaf and fruit metabolic profiles are coordinated or follow distinct, organ‐specific trajectories remains poorly understood. Moreover, leaves are commonly regarded as metabolically plastic organs, as their physiology and metabolism are strongly influenced by external factors, such as light, temperature, and biotic interactions. Consequently, whether stable, genotype‐associated organization of specialized metabolic pathways can be detected in leaf tissues remains an open question.

In this study, we combined untargeted metabolomic profiling with absolute quantification of major phenylpropanoids to compare leaves and fruits of sweet cherry cultivars at full fruit maturity. Our objective was (i) to investigate tissue‐specific metabolic channels within the phenylpropanoid pathway and (ii) to determine whether phenylpropanoid pathway organization in leaves exhibits a reproducible genotype‐associated structure across different orchards and growing seasons, analogous to that previously observed in fruits, thereby extending the analysis of genotype‐associated metabolic organization to a vegetative tissue traditionally regarded as highly plastic.

## 2 Materials and Methods

### 2.1 Experimental Design and Plant Material Collection

**FIGURE 1:**
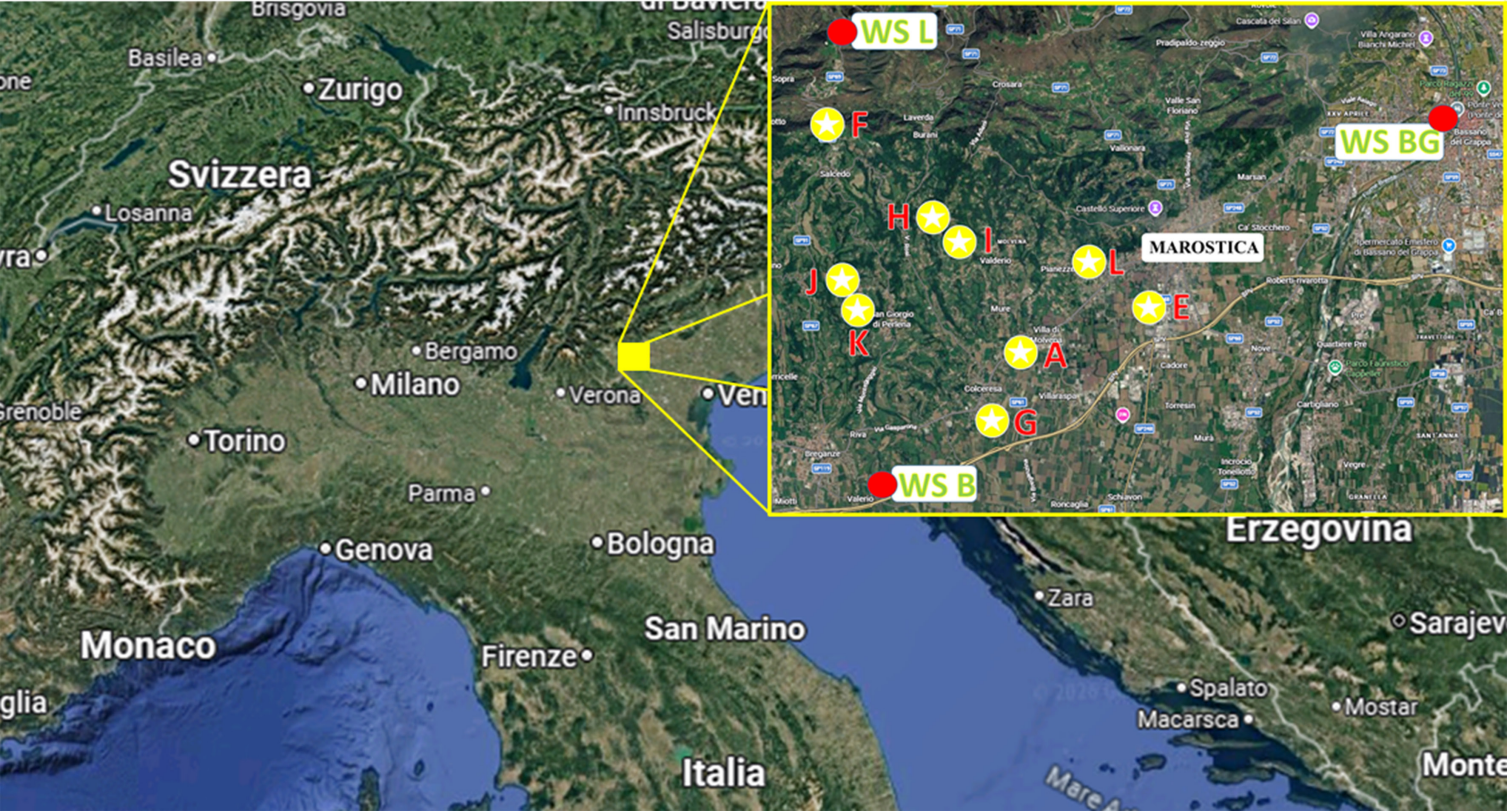
Geographical distribution of sampled orchards and meteorological stations within the Marostica PGI production area.

Plant material and sampling were performed following the experimental design described in Commisso et al. (2017), with minor modifications. In particular, each orchard was considered as an independent sampling unit. Fully mature fruits and corresponding leaves of *Prunus avium* L. were collected from seven cultivars, including early‐ (Early Bigi, Burlat, and Sandra), mid‐ (Grace Star and Romana), and late‐ripening (Durone Rosso and Ferrovia) cultivars during the 2014 and 2015 seasons within the Marostica PGI production area (Vicenza, Italy; Figure 1). For each cultivar, material was obtained from three to four distinct orchards, based on availability (Table S1). Within each orchard, sampling was performed on up to 20 trees when possible. Meteorological data representative of the sampled production area were compiled from three weather stations located in Lusiana, Breganze, and Bassano del Grappa and are reported in Table S2. In particular, monthly rainfall, number of rainy days, solar radiation, and mean temperature were compiled for each station and year. Approximately 50 fruits and 20 leaves were harvested per tree, ensuring that samples originated from multiple canopy positions and from trees of different ages in order to capture intra‐orchard variability. After collection, plant material was placed in labeled paper bags and transferred to the laboratory. On the day of harvest, fruits and leaves collected from individual trees were randomly combined to form pooled samples comprising either 50 fruits or 20 leaves. This pooling process was carried out independently three times, generating three biological replicates for subsequent metabolomic analyses. For metabolite profiling, fruits were manually destoned using a commercial cherry pitter (Westmark) and longitudinally divided into four equal segments. Two segments per fruit were immediately frozen in liquid nitrogen and used to establish technical replicates corresponding to each biological replicate, while an additional replicate was retained as backup material. Leaves were manually detached from the petiole and immediately frozen in liquid nitrogen. Frozen samples were then finely ground under liquid nitrogen using an A11 basic analytical mill (IKA‐Werke), and the resulting powders were stored at −80°C until further analysis. Fruit quality parameters were assessed using a separate composite sample of 30 fruits. Fruit firmness was measured at four evenly spaced points on each fruit with a handheld sclerometer (Agro Technologies), and individual fruit weight was recorded. Juice pH was determined using a digital pH meter (microPH 2001, Crison), while soluble solid content was measured as °Brix using a digital refractometer (DBR 35, Ningbo Kingstic). Leaf fresh weight was determined using three independent composite samples, each consisting of 20 leaves.

### 2.2 Extraction of Phenolic Compounds From Fruit and Leaf Tissues

Frozen powdered fruit and leaf tissues were extracted using an acidified chloroform–methanol solvent system, consisting of LC–MS‐grade chloroform and methanol (2:1, v/v; Honeywell) supplemented with 1% (v/v) hydrochloric acid prepared from a 37% stock solution. Specifically, 300 mg of fruit powder and 150 mg of leaf powder were extracted with solvent volumes corresponding to 10‐fold and 30‐fold (w/v), respectively. Samples were vortex‐mixed for 1 min, after which LC–MS‐grade water (Honeywell) was added in volumes corresponding to 2‐fold (w/v) for fruit extracts and 6‐fold (w/v) for leaf extracts, relative to the initial tissue powder, to induce phase separation. The mixtures were subsequently sonicated for 15 min at 40 kHz in an ultrasonic bath (Falc Instruments) and centrifuged at 4500 × *g* for 10 min at 4°C. Following centrifugation, the upper hydroalcoholic phase was carefully transferred to 2 mL microcentrifuge tubes and subjected to a second centrifugation step at 21,000 × *g* for 10 min at 4°C to remove residual particulate material. The clarified supernatants were then collected and stored at −20°C until analysis.

### 2.3 Chromatographic Quantification of Major Phenolics by HPLC–DAD

Polyphenolic compounds were quantified by HPLC coupled to diode array detection following an established analytical workflow (Commisso et al. 2017), adapted for the present study. Prior to chromatographic analysis, fruit and leaf methanolic extracts were diluted tenfold with LC–MS‐grade water (Honeywell) and clarified by filtration through 0.2 μm Minisart RC4 syringe filters (Sartorius Stedim). Injection volumes were set to 40 μL for fruit extracts and 20 μL for leaf extracts. Analyses were performed using a Beckman Coulter liquid chromatography system equipped with a Gold 126 solvent delivery module, a Gold 168 diode array detector, and a System Gold 508 autosampler (Beckman Coulter). Chromatographic separation was achieved using a reversed‐phase gradient composed of solvent A (LC–MS‐grade water containing 0.5% v/v formic acid and 5% v/v acetonitrile) and solvent B (100% acetonitrile). The gradient program consisted of an initial increase from 0% to 10% B over 2 min, followed by a rise to 20% B over 10 min, then to 25% B over 2 min, and to 70% B over 7 min. This was followed by an isocratic step at 70% B for 5 min, a rapid increase to 90% B in 1 min, an isocratic hold at 90% B for 14 min, and a return to initial conditions within 1 min. Column re‐equilibration was performed for 18 min prior to the next injection. Separation was carried out on an Alltima HP C18 analytical column (150 × 2.1 mm, 3 μm particle size) preceded by a C18 guard column (7 × 2.1 mm), both supplied by Alltech Associates. The flow rate was maintained at 0.2 mL min^−1^ throughout the analysis. Chromatographic data acquisition and processing were conducted using 32 Karat Workstation software (version 7.0; Beckman Coulter). To support the identification of the main peaks detected by DAD, additional UPLC–HRMS analyses were performed specifically for peak assignment. These analyses were conducted using the UPLC–HRMS instrumentation described below, but applying the same stationary phase, chromatographic gradient, and flow rate adopted for the HPLC–DAD method, thereby enabling partial alignment of retention times between the two analytical platforms. These targeted UPLC–HRMS analyses were used exclusively for the identification of DAD‐detected compounds, whereas untargeted metabolomic profiling was carried out using a distinct UPLC–HRMS method, described in the corresponding section of the Materials and Methods. Quantification was based on external calibration curves constructed from serial dilutions of authentic reference compounds (*R* ^2^ ≥ 0.999), including 3‐caffeoylquinic acid (chlorogenic acid), quercetin‐3‐O‐glucoside (isoquercetin), kaempferol‐3‐O‐rutinoside (nicotiflorin), cyanidin‐3‐O‐glucoside (kuromanin), p‐coumaric acid, and (+)‐catechin (Extrasynthese). Detection wavelengths were selected according to compound class: 320 nm for hydroxycinnamic acids, 350 nm for flavonols, 280 nm for flavan‐3‐ols, and 520 nm for anthocyanins. Metabolites for which authentic standards were not available were quantified using structurally related compounds as equivalents. Accordingly, neochlorogenic acid and caffeoyl quinic acid methyl ester were expressed as 3‐caffeoylquinic acid equivalents; p‐coumaroylquinic acid as p‐coumaric acid equivalents; quercetin rutinosylated derivatives as quercetin‐3‐O‐glucoside equivalents; kaempferol glycosylated and acylated derivatives as kaempferol‐3‐O‐rutinoside equivalents; and anthocyanins as cyanidin‐3‐O‐glucoside equivalents.

### 2.4 UPLC–HRMS Profiling of Fruit and Leaf Metabolomes

Untargeted high‐resolution mass spectrometry (HRMS) analyses were conducted using an ultra‐performance liquid chromatography (UPLC) platform coupled to quadrupole time‐of‐flight detection, based on previously published methodologies (Fasani et al. 2025) with minor adjustments. Prior to injection, methanolic extracts obtained from fruit and leaf samples were diluted with LC–MS‐grade water (Honeywell) at ratios of 1:10 and 1:50, respectively. Chromatographic separation was achieved using an ACQUITY I‐Class UPLC system (Waters) interfaced with a Xevo G2‐XS QqTOF mass spectrometer (Waters) equipped with an electrospray ionization source operating in both positive and negative ionization modes. Instrument control, data acquisition, and processing were performed using MassLynx software (version 4.1, Waters). Samples were injected onto an ACQUITY UPLC HSS T3 reversed‐phase column (2.1 × 100 mm, 1.8 μm; Waters), which was maintained at 30°C throughout the analysis. The mobile phase consisted of solvent A (water containing 0.1% formic acid; Biosolve Chimie) and solvent B (acetonitrile), both of LC–MS grade (Honeywell). Elution was performed at a constant flow rate of 0.35 mL min^−1^ and using the following gradient: 1% B as initial condition, held to 1% B for 1 min, then increased to 40% B at 10 min, to 70% B at 13.5 min, and to 99% at 14 min. Subsequently, the method remained in 99% B for 2 min and was then decreased to 1% B at 16.1 min. The column was subsequently re‐equilibrated at starting conditions, yielding a total run time of 20 min per sample. Samples were maintained at 8°C in the autosampler, and injection order was randomized. To monitor analytical reproducibility and instrument performance, a pooled quality control (QC) sample was prepared by combining equal aliquots of methanolic extracts and was injected at regular intervals (every nine samples). In addition, system stability was assessed using a standard solution containing chlorogenic acid, daidzein, and naringenin (1 ng μL^−1^), phenylalanine (2 ng μL^−1^), and gallic acid, quercetin, and dehydroartemisininic acid (3 ng μL^−1^). One microliter of this mixture was injected immediately after each QC run. Mass spectrometric parameters were set as follows: capillary voltage 0.8 kV, sampling cone voltage 40 V, source offset voltage 80 V, source temperature 120°C, and desolvation temperature 500°C. Nitrogen was used as nebulizing and desolvation gas at flow rates of 50 and 1000 L h^−1^, respectively, while argon served as collision gas. Data were acquired in positive and negative ionization modality, and the chromatograms acquired in negative ionization mode were used to build the subsequent datamatrix, while positive analyses were used to support the metabolite identification process. The acquisition was performed in continuum mode using two scan functions with fixed collision energies. The first function was acquired without collision energy, whereas the second function was operated at 35 V. For a subset of samples, collision energy settings were adjusted to optimize fragmentation, increasing the energy to 45 V or decreasing it to 20 V depending on the metabolites of interest. In addition, a FAST‐DDA acquisition strategy was implemented to improve metabolite identification efficiency (Zorzi et al. 2023). Data were acquired in sensitivity mode between 1.4 and 14 min over a mass range of 50–2000 m/z, using a scan time of 0.3 s. Mass accuracy was maintained through continuous lock‐mass correction using a leucine‐enkephalin solution (100 pg μL^−1^; Waters) infused at 10 μL min^−1^, providing reference ions at m/z 556.2771 (positive mode) and 554.2615 (negative mode).

### 2.5 Computational Processing and Annotation of Metabolomic Data

Untargeted metabolomic data files obtained by using negative ionization mode were processed using Progenesis QI software (Waters) according to the default data processing workflow. Following feature alignment and peak detection, the resulting data matrix was normalized by converting metabolite signal intensities into relative abundance values. For each sample, the sum of all detected signals was set to 100%, and individual metabolite abundances were expressed as percentages of the total signal, thereby allowing comparison of relative metabolite composition among samples and organs. Following data matrix generation, an additional manual post‐processing was applied and m/z features detected in solvent blank samples were excluded prior to statistical analysis. Preliminary metabolite annotation was performed through automated matching against publicly available databases, including MassBank, PlantCyc, the Plant Metabolic Network, and the Human Metabolome Database, based on accurate mass measurements, isotopic patterns, and fragmentation spectra generated during data processing. Additional annotation was achieved by querying the Metlin database (https://metlin.scripps.edu) using a mass tolerance of 0.003 Da and by comparison with an in‐house library of authentic reference standards. Published literature was further consulted to support metabolite assignment. All proposed metabolite identifications were subsequently confirmed by manual inspection of the spectral data and the annotation confidence reported as described in Sumner et al. (2007).

### 2.6 Exploratory and Discriminant Statistical Analyses

The feature matrix generated by Progenesis QI, containing m/z values and relative metabolite abundances, was subjected to multivariate statistical analysis. Principal component analysis (PCA) was performed using SIMCA software version 13.0 (Umetrics) after mean‐centering and Pareto scaling to explore intrinsic data structure and sample clustering. Samples falling outside the Hotelling's *T* ^2^ confidence region or showing elevated DModX values were considered outliers and removed prior to multivariate analysis. Supervised multivariate analyses were subsequently conducted by assigning sample groupings as response variables (*Y*) and applying orthogonal partial least‐squares discriminant analysis (OPLS‐DA) or orthogonal bidirectional partial least‐squares discriminant analysis (O2PLS‐DA), depending on the specific comparison and experimental design. Partial least‐squares discriminant analysis (PLS‐DA) was additionally performed to support model validation, and PLS‐DA models were evaluated using permutation testing (400 permutations). Model validity for OPLS‐DA and O2PLS‐DA analyses was further assessed by cross‐validation followed by analysis of variance (CV‐ANOVA), using a significance threshold of *p* < 0.01. For OPLS‐DA and O2PLS‐DA models, metabolites contributing most strongly to class separation were identified based on pq(corr) values, which describe the correlation between metabolite variables (*X* matrix) and class membership (*Y* matrix). To further support multivariate results, univariate statistical analyses were performed on selected metabolites using Students *t*‐test (*p* < 0.01) or one‐way analysis of variance (ANOVA) followed by Tukeys post hoc test, with statistical significance defined as *p* < 0.05. Heat maps were used to visualize the relative abundance of metabolites selected from the O2PLS–DA loading plots as the variables most associated with class discrimination. No hierarchical clustering was applied to either samples or metabolites. Metabolites were arranged according to their class association in the corresponding loading plots, and cell colors represent normalized relative abundance values.

## 3 Results

### 3.1 Agronomic and Meteorological Context of the Sampled Material

Fruits and leaves from seven *Prunus avium* cultivars were collected across nine orchards during the 2014 and 2015 growing seasons, at the stage of commercial fruit maturity as determined in collaboration with orchard managers (Table S1). To provide an environmental context for the sampled material, meteorological data from three weather stations representative of the production area were compiled for the 2014 and 2015 growing seasons (Table S2). These data showed year‐associated meteorological differences, particularly when annual parameters were considered. Overall, 2014 was characterized by higher total rainfall and a higher number of rainy days, whereas 2015 showed lower rainfall, fewer rainy days, and higher solar radiation. However, climatic differences during key phenological periods, including flowering and harvest, were less pronounced than those observed on an annual basis. Thus, the metabolomic dataset was obtained under contrasting annual meteorological conditions. This environmental information was used to contextualize the interpretation of metabolomic patterns across cultivars, years, and orchard locations. Agronomic assessment confirmed that fruits were harvested at comparable physiological maturity, as reflected by similar values of fruit weight, firmness, total soluble solids, and pH across cultivars. As the present study aims to investigate leaf and fruit metabolomes, agronomic traits are not further discussed.

### 3.2 Untargeted Metabolomics Revealed the Specialized Metabolites Occurring in Fruits and Leaves

**FIGURE 2:**
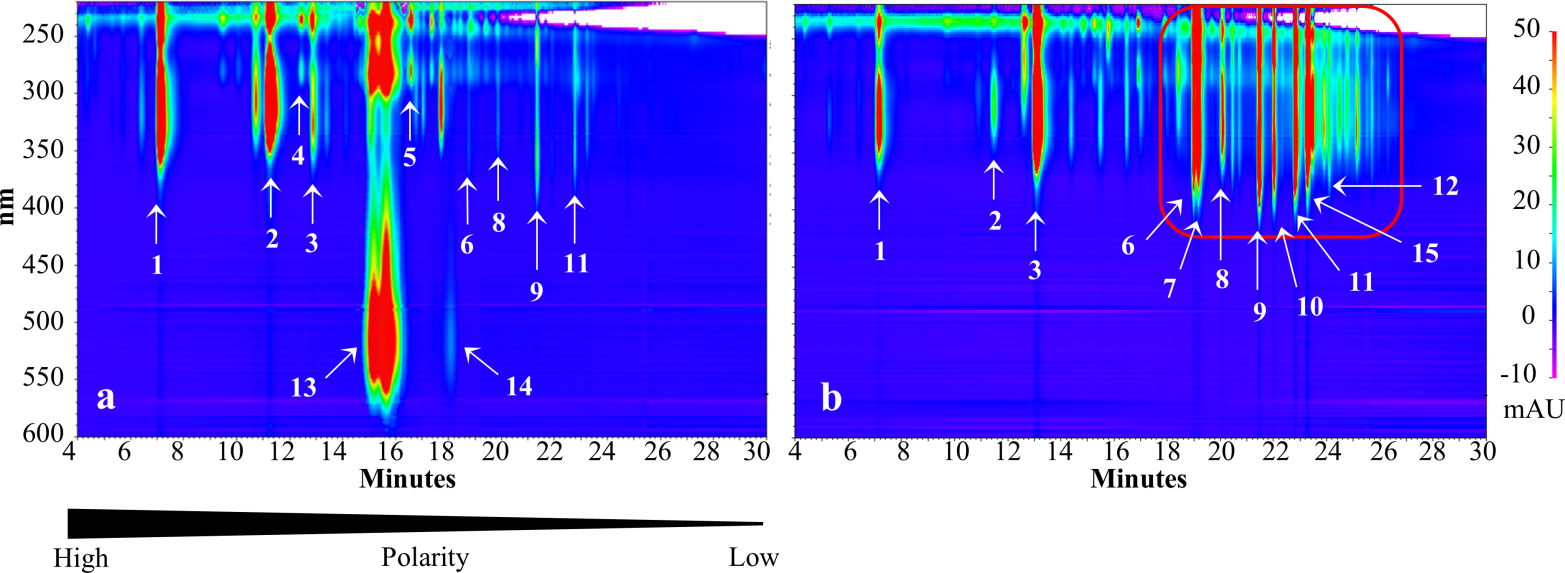
HPLC–DAD chromatographic profiles of representative sweet cherry fruit (a) and leaf (b) extracts. Chromatograms were recorded in the 280–600 nm range. The color bar indicates absorbance intensity (mAU, arbitrary units), reflecting metabolite abundance in the extracts. Fruit extracts are dominated by mildly to highly polar metabolites, whereas leaf extracts show a higher contribution of less polar compounds (highlighted area). Peak numbers correspond to metabolites identified by UPLC–HRMS and reported in Table 1.

Untargeted metabolomic profiling revealed a clear and expected organ‐specific differentiation between fruits and leaves of sweet cherry. HPLC–DAD chromatograms showed that fruit extracts were dominated by mildly to highly polar metabolites (Figure 2a), whereas leaf extracts displayed a prevalence of less polar compounds (Figure 2b).

The major metabolites detected by HPLC–DAD were identified by UPLC–HRMS and mainly belonged to hydroxycinnamic acids, flavonols, flavan‐3‐ols, and anthocyanins (Table 1). While most compounds were detected in both organs, several metabolites showed a marked organ preference, with anthocyanins predominantly detected in fruits and flavonol derivatives, particularly kaempferol‐O‐acetylhexoside and kaempferol‐O‐malonylhexoside, enriched in leaves. In addition, genotype‐dependent accumulation patterns were observed.

**TABLE 1:** Metabolite content in fruits and leaves of different cultivars (mg/100 g fresh weight).

	Leaf						
	Sandra	Brulat	Romana	Durone Rosso	Ferrovia	Early Bigi	Grace Star
1	188.84 ± 30.80a	95.17 ± 10.15c,d	102.38 ± 17.84b,c	68.32 ± 5.78d,e	68.63 ± 9.87d,e	49.15 ± 16.45e	123.22 ± 10.57b
2	64.42 ± 11.42a	34.95 ± 6.79b	82.04 ± 18.58a	27.55 ± 8.63b,c	43.71 ± 17.68b	12.58 ± 11.77c	7.81 ± 1.42c
3	252.38 ± 62.62a	341.56 ± 64.77a	342.56 ± 137.05a	202.75 ± 34.39a	247 0.40 ± 33.08a	318.60 ± 142.10a	272.46 ± 110.35a
4	− ± −	− ± −	− ± −	− ± −	− ± −	− ± −	− ± −
5	− ± −	− ± −	− ± −	− ± −	− ± −	− ± −	− ± −
6	59.61 ± 7.85a	66.78 ± 7.00a	41.75 ± 4.80b	34.56 ± 3.56b	32.60 ± 3.20b	57.47 ± 8.46a	56.11 ± 10.10a
7	115.39 ± 16.25a	106.00 ± 12.35ab	95.63 ± 12.15b	55.99 ± 5.04c	53.54 ± 6.40c	95.03 ± 13.40b	92.10 ± 11.17B
8	28.33 ± 6.20a	37.11 ± 8.07a	37.91 ± 14.12a	22.97 ± 4.48a	28.39 ± 4.59a	36.86 ± 15.45a	30.43 ± 11.60a
9	113.67 ± 18.46b	153.70 ± 14.98a	69.93 ± 16.46c	76.09 ± 10.20c	78.65 ± 8.53c	118.95 ± 30.62b	98.03 ± 26.71b,c
10	61.87 ± 10.12a,b	72.39 ± 10.29a	32.22 ± 8.25c	43.98 ± 11.01b,c	59.93 ± 17.26a,b	54.27 ± 17.39a,b	44.79 ± 13.06b,c
11	160.98 ± 16.50a	159.85 ± 18.25a	145.58 ± 27.61a,b,c	111.12 ± 11.76c	114.75 ± 6.43bc	149.13 ± 35.56ab	127.59 ± 22.85abc
12	2.39 ± 0.82c	1.02 ± 2.87c	11.31 ± 3.40a,b	11.49 ± 3.17ab	15.23 ± 3.53a	9.88 ± 3.38b	8.61 ± 1.45b
13	− ± −	− ± −	− ± −	− ± −	− ± −	− ± −	− ± −
14	− ± −	‐ ± −	− ± −	− ± −	− ± −	− ± −	− ± −

	Fruit						
	Sandra	Brulat	Romana	Durone Rosso	Ferrovia	Early Bigi	Grace Star
1	37.47 ± 5.64a	15.78 ± 3.83c	22.80 ± 3.96b	13.81 ± 1.99c	17.83 ± 4.38b,c	6.58 ± 1.67d	34.41 ± 5.59a
2	11.15 ± 2.07b	18.76 ± 5.27a	19.30 ± 4.48a	9.94 ± 1.77b,c	9.85 ± 2.58b,c	5.73 ± 2.51c	5.35 ± 1.68c
3	3.03 ± 0.46b,c	3.71 ± 0.80a,b	4.30 ± 0.81a	3.20 ± 0.52a,b,c	3.45 ± 0.57a,b,c	2.66 ± 0.73c	3.67 ± 0.67a,b
4	5.47 ± 1.07a	5.72 ± 1.29a	3.21 ± 0.95b	2.42 ± 0.74b	2.48 ± 0.51b	3.79 ± 0.78b	2.47 ± 0.21b
5	4.16 ± 1.72c	7.40 ± 2.08a,b	8.02 ± 1.61a	4.69 ± 0.80c	5.86 ± 0.40a,b,c	5.27 ± 1.78b,c	6.21 ± 1.60a,b,c
6	*16.41 ± 1.04a	*15.66 ± 0.99ab	*13.57 ± 0.68b	*16.56 ± 1.77a	*16.43 ± 2.54a	*15.70 ± 1.73ab	*13.61 ± 0.53b
7	*6.91 ± 0.73a	*6.38 ± 0.30a	*6.26 ± 0.54a	*7.84 ± 1.23a	*7.54 ± 1.30a	*5.58 ± 0.70b	*5.98 ± 0.73a
8	*3.37 ± 0.42a	*3.80 ± 0.85a	*3.94 ± 0.80a	*3.31 ± 0.66a	*3.36 ± 0.48a	*3.11 ± 0.77a	*3.86 ± 0.92a
9	3.81 ± 0.92a	3.54 ± 0.77a	3.29 ± 0.57a	3.79 ± 0.68a	3.45 ± 0.76a	3.82 ± 0.95a	2.77 ± 0.56a
10	*20.37 ± 8.15a	*19.65 ± 12.92a	*11.01 ± 0.82a	*12.32 ± 2.30a	*10.91 ± 0.98a	*16.36 ± 4.25a	*9.62 ± 1.85a
11	*9.65 ± 1.15b	*8.87 ± 0.84b	*15.03 ± 3.41a	*11.85 ± 2.93b	*12.21 ± 2.53b	*8.02 ± 1.92b	*9.86 ± 2.54b
12	− ± −	− ± −	− ± −	− ± −	− ± −	− ± −	− ± −
13	60.69 ± 23.88a,b	65.10 ± 31.26a,b	31.67 ± 7.52b	64.03 ± 17.42a,b	48.13 ± 18.58ab	71.39 ± 23.42a	34.39 ± 11.40b
14	1.14 ± 0.43a,b	1.30 ± 0.64a,b	1.32 ± 0.65a,b	1.54 ± 0.74ab	1.02 ± 0.59b	2.03 ± 0.65a	1.54 ± 0.62a,b

*Note:* Peaks: neochlorogenic acid (1), coumaroyl quinic acid 1 (2), chlorogenic acid (3), (+)‐catechin (4), (−)‐epicatechin (5), quercetin‐O‐deoxyhexosyl hexosyl hexoside (6), caffeoyl quinic acid methyl ester (8), quercetin‐3‐O‐rutinoside (9), quercetin‐3‐O‐glucoside (10), kaempferol‐3‐O‐rutinoside (11), kaempferol‐O‐acetylhexoside plus kaempferol‐O‐malonylhexoside (12), cyanidin‐3‐O‐glucoside plus cyanidin‐3‐O‐rutinoside (13), and peonidin‐3‐O‐rutinoside (14). (−) Not quantified. Letters indicate significance differences among groups after one‐way ANOVA and HSD Tukeys post Hoc test, *p* < 0.05.

* Values expressed as mg/kg FW.

In fruits, genotype‐dependent differences mainly involved the accumulation of a small number of phenolic compounds (Table 1). Early Bigi showed the highest levels of cyanidin derivatives, while Romana and Grace Star displayed consistently lower amounts. Conversely, neochlorogenic acid accumulated preferentially in Sandra and Grace Star and was less represented in Early Bigi, whereas coumaroyl quinic acid was enriched in Burlat and Romana and reduced in Early Bigi and Grace Star.

In leaves, genotype‐dependent variation mainly affected the accumulation of hydroxycinnamic acids and flavonols (Table 1). Neochlorogenic acid showed marked cultivar specificity, with the highest levels detected in Sandra and the lowest in Early Bigi. Similarly, kaempferol‐3‐O‐rutinoside was most abundant in Sandra and Burlat and comparatively reduced in Durone Rosso. In contrast, chlorogenic acid did not display significant differences among cultivars, despite being one of the most abundant leaf metabolites.

The observed accumulation patterns were consistent across the two growing seasons (Table S3). Conversion of metabolite content from a fresh‐weight basis to a per‐organ basis resulted in only minor changes in cultivar ranking, and the overall trends remained consistent (Table S4). Across all cultivars, fruits consistently accumulated higher levels of neochlorogenic acid than chlorogenic acid, whereas the opposite trend was observed in leaves.

### 3.3 UPLC–HRMS Profiling Highlights Organ‐Specific Metabolite Class Distribution

**FIGURE 3:**
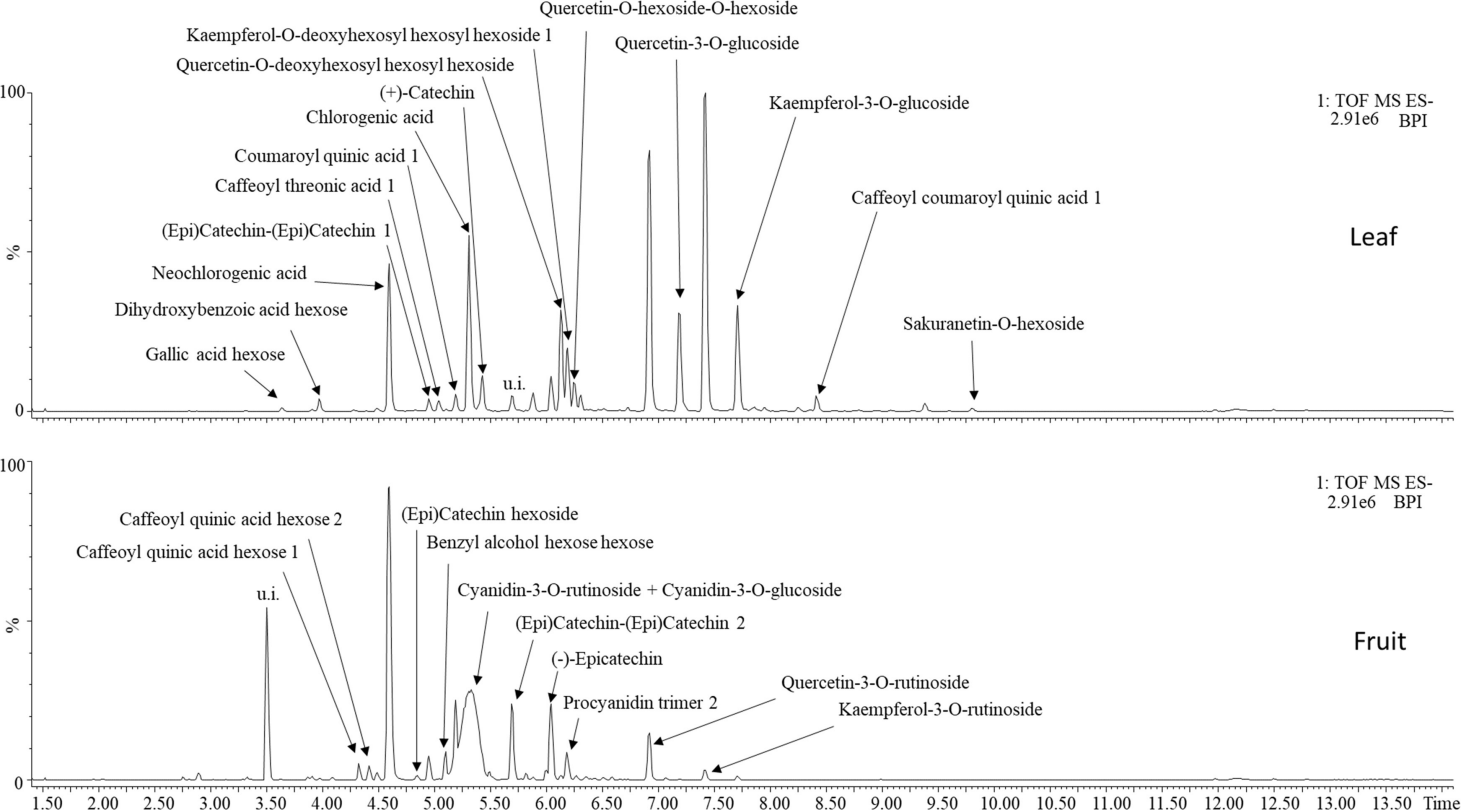
UPLC–HRMS base peak chromatograms of representative sweet cherry fruit and leaf extracts. Chromatograms highlight the organ‐specific distribution of major metabolites. Peak identities are reported where available; U.i., unidentified compound. Time is expressed in minutes. BPI, Base Peak Ion.

UPLC–HRMS‐based untargeted metabolomics further confirmed the strong differentiation between fruits and leaves, generating two distinct base peak chromatographic profiles depending on the analysed organ (Figure 3).

**FIGURE 4:**
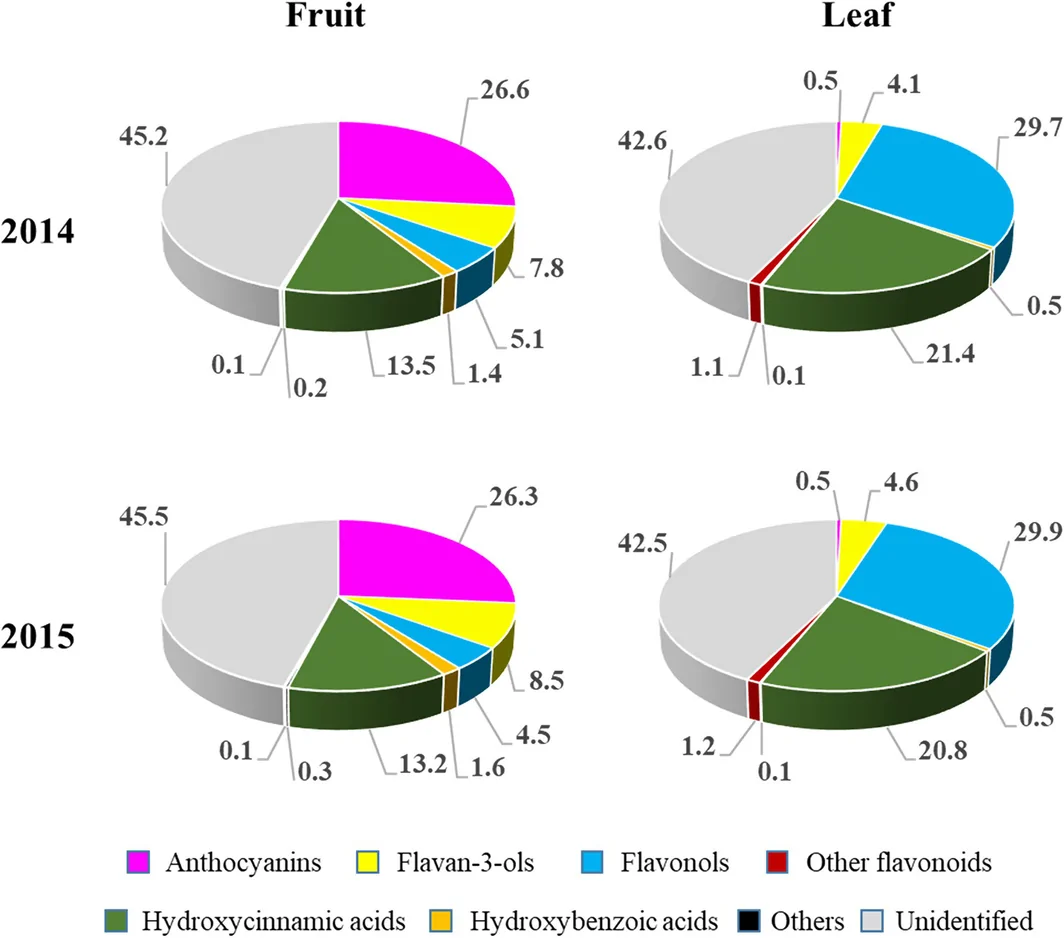
Relative distribution of metabolite classes in fruit and leaf extracts of sweet cherry obtained by UPLC–HRMS analysis. Values are expressed as percentage of the total ion signal (set to 100%) and represent the mean ± standard deviation of all cultivars in the two growing seasons (2014 and 2015). HCAs, Hydroxycinnamic acids; HBAs, Hydroxybenzoic acids.

Data processing resulted in a matrix of 3219 signals, of which 115 m/z features were putatively annotated (File S1). These metabolites encompassed diverse chemical classes, including hydroxycinnamic acids, hydroxybenzoic acids, flavonoids, anthocyanins, flavan‐3‐ols, flavanones, flavones, chalcones, flavonolignans, cyanogenic glycosides, jasmonates, and stilbenoids (Figure 4).

The annotated metabolites accounted for approximately 54%–57% of the total chromatographic signal, and their distribution markedly differed between organs (Figure 4). Leaves were mainly characterized by flavonols (~29% total signal), hydroxycinnamic acids (~21% total signal), and flavan‐3‐ols (~4% total signal), whereas fruits were dominated by anthocyanins (~26% total signal), followed by hydroxycinnamic acids (~13% total signal), flavan‐3‐ols (~8% total signal), and flavonols (~4% total signal). Comparable metabolite class distributions were observed across the two growing seasons.

### 3.4 Multivariate Analysis Discriminates Fruits and Leaves Based on Metabolite Composition

**FIGURE 5:**
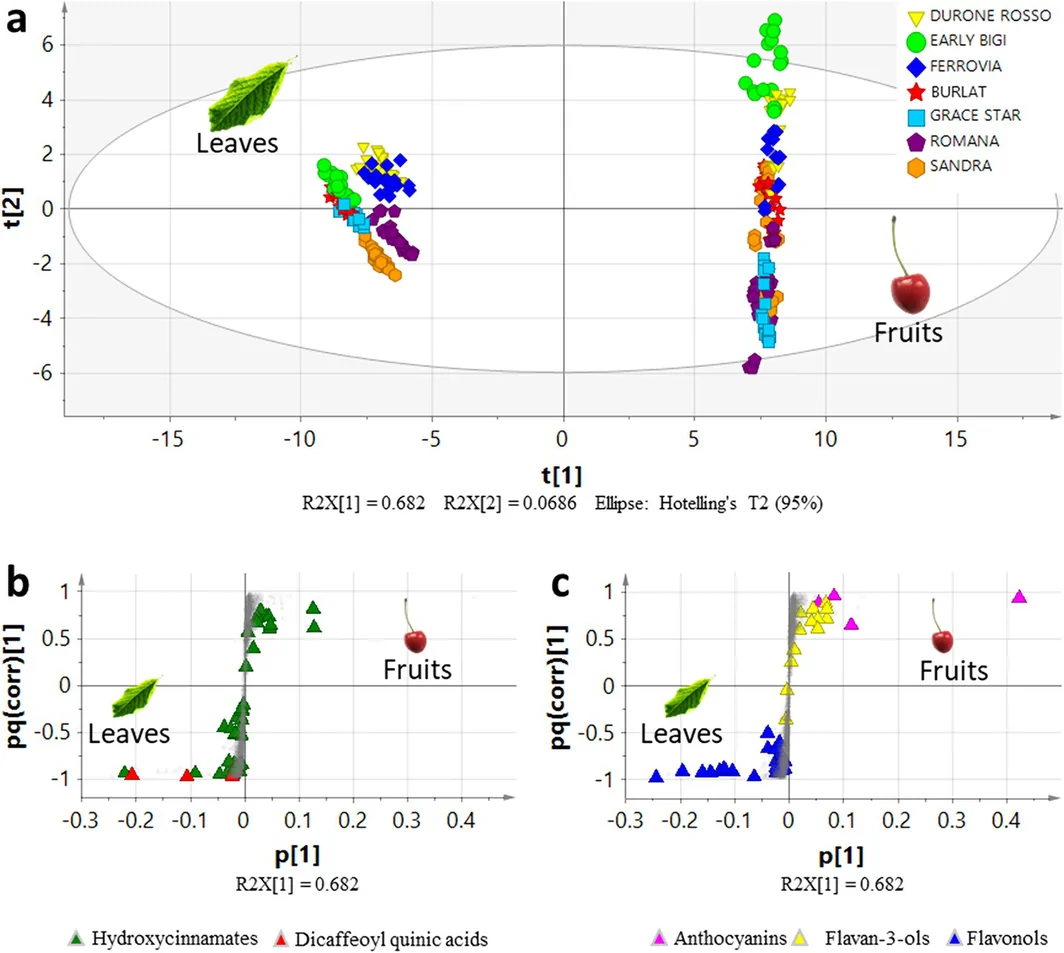
Multivariate statistical analysis of fruit and leaf metabolomic profiles. (a) Score scatter plot of principal component analysis (PCA) performed on the UPLC–HRMS dataset, showing clear separation between fruit and leaf samples along PC1. (b, c) OPLS–DA loading plots obtained by comparing fruit and leaf samples, illustrating metabolites positively correlated with each organ. Triangles represent metabolites, and colors indicate the metabolite chemical class.

Multivariate analysis of the UPLC–HRMS dataset revealed a clear and expected separation between fruit and leaf samples. Principal component analysis (PCA) showed a strong organ‐driven discrimination, with the first principal component explaining 68.2% of the total variance (Figure 5a).

Supervised OPLS–DA analysis further highlighted the metabolites most strongly associated with each organ (Figure 5b,c; File S2). Hydroxycinnamic acid derivatives, particularly dicaffeoylquinic acid isomers, and flavonols were mainly associated with leaf samples, whereas anthocyanins and flavan‐3‐ols were preferentially associated with fruits.

### 3.5 Genotype‐Dependent Variation in Fruit and Leaf Metabolome Composition

**FIGURE 6:**
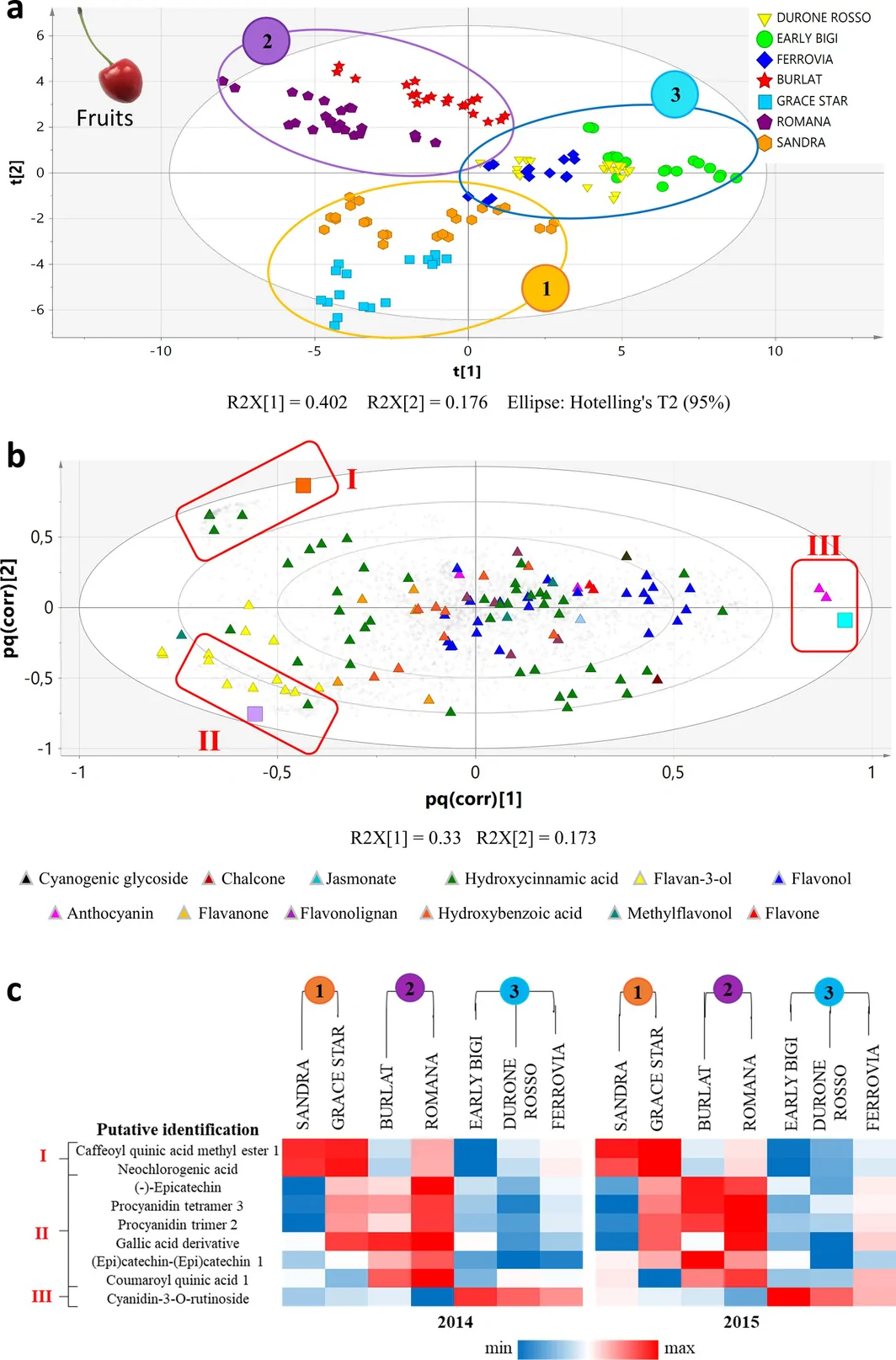
Multivariate statistical analysis of sweet cherry fruit metabolomes. (a) PCA score scatter plot showing cultivar‐specific clustering of fruit samples and the identification of three main cultivar groups. (b) O2PLS–DA loading plot highlighting metabolites associated with each cultivar group. Triangles represent metabolites, and boxes represent classes. Different colors were used to highlight the chemical class of the metabolite. (c) Heat map showing relative abundances of fruit metabolites selected from the O2PLS–DA loading plot as the variables most associated with cultivar‐group discrimination. No hierarchical clustering was applied; metabolites are arranged according to their association with the corresponding cultivar groups. Colors indicate normalized abundance levels (red, high; blue, low).

To investigate genotype effects independently within each organ, fruit and leaf datasets were analysed separately. PCA of fruit samples explained 57.8% of the total variance across the first two components and revealed a cultivar‐associated distribution of samples (Figure 6a). The overall PCA model included six components and showed good descriptive and predictive performance (*R* ^2^ *X*(cum) = 0.869; *Q* ^2^(cum) = 0.812). Although the dataset included samples collected for each cultivar across two different growing seasons and from multiple commercial orchards, samples belonging to the same cultivar consistently clustered together.

Based on metabolome similarity, three main cultivar‐groups were identified: Sandra and Grace Star (group 1), Romana and Burlat (group 2), and Durone Rosso, Ferrovia, and Early Bigi (group 3). Subsequent O2PLS–DA analysis identified the metabolites contributing most strongly to the discrimination among cultivar groups (Figure 6b,c). The cultivar‐based model, built using three cultivar groups as class labels, included two predictive and three orthogonal components and showed high descriptive and predictive performance (*R* ^2^ *X*(cum) = 0.829; *R* ^2^ *Y*(cum) = 0.900; *Q* ^2^(cum) = 0.888), supporting the presence of a strong cultivar‐associated component in fruit metabolomic profiles. Sandra and Grace Star were positively characterized by feruloyl quinic acid isomers and neochlorogenic acid, whereas Romana and Burlat were associated with flavan‐3‐ols and coumaroyl quinic acid derivatives. The third group, including Durone Rosso, Ferrovia, and Early Bigi, was mainly characterized by anthocyanins, particularly cyanidin‐3‐O‐rutinoside.

To explicitly evaluate whether this distribution was associated with growing season or orchard of origin, the same fruit PCA score plot was additionally visualized by coloring samples according to year and orchard (Figure S1a,d). Compared with the cultivar‐based representation, no clear separation exclusively associated with year or orchard was observed, suggesting that cultivar‐associated structure was more evident than year‐ or orchard‐associated separation in the unsupervised analysis.

Because the two growing seasons differed in meteorological conditions, a supervised OPLS–DA model was built to evaluate the year effect in fruit samples (Figure S1b,c). The model separated samples according to growing season and showed high predictive ability (*R* ^2^ *X*(cum) = 0.902; *R* ^2^ *Y*(cum) = 0.901; *Q* ^2^(cum) = 0.847), indicating that inter‐annual environmental variation contributed to the fruit metabolome. However, this year‐associated separation was not evident in the unsupervised PCA, and the predictive component accounted for only a small fraction of the *X* variance (*R* ^2^ *X*[1] = 0.023), suggesting that the year effect, although detectable by supervised modeling, was not the most evident source of variation in the unsupervised fruit metabolomic dataset.

The possible contribution of orchard location was also evaluated by O2PLS–DA using the nine orchards as class labels (Figure S1e,f). The model explained a large fraction of the *X* variance (*R* ^2^ *X*(cum) = 0.908), but only a moderate fraction of the *Y* variance (*R* ^2^ *Y*(cum) = 0.413), with limited predictive ability (*Q* ^2^(cum) = 0.310). Consistently, the score plot did not show clear orchard‐specific clustering, and the corresponding loading plot did not reveal strong orchard‐specific metabolite associations. These results suggest that orchard‐related variation was detectable but less clearly structured than cultivar‐associated variation.

**FIGURE 7:**
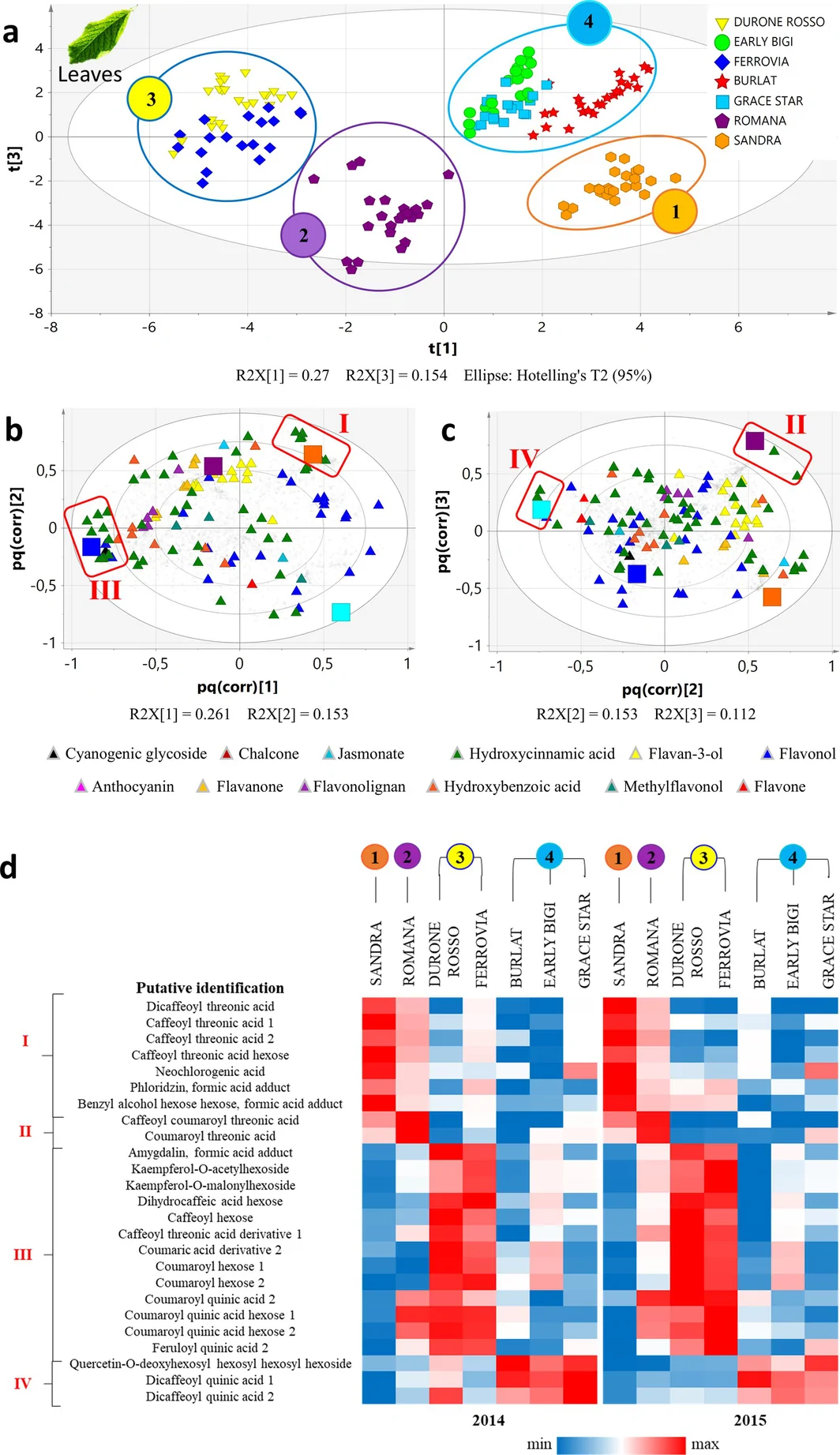
Multivariate statistical analysis of sweet cherry leaf metabolomes. (a) PCA score scatter plot showing cultivar‐dependent clustering and the identification of four main groups. (b, c) O2PLS–DA loading plots displaying metabolites correlated with specific cultivar groups. Triangles represent metabolites, and boxes represent classes. Different colors were used to highlight the metabolite chemical class. (d) Heat map showing relative abundances of leaf metabolites selected from the O2PLS–DA loading plots as the variables most associated with cultivar‐group discrimination. No hierarchical clustering was applied; metabolites are arranged according to their association with the corresponding cultivar groups. Colors indicate normalized abundance levels (red, high; blue, low).

PCA of leaf samples revealed a clear cultivar‐dependent clustering (despite, as for fruits, samples of each cultivar originating from different orchards and being collected across two growing seasons), identifying four distinct groups (Figure 7a). Sandra and Romana each formed independent clusters, while Durone Rosso and Ferrovia grouped together, and Burlat, Grace Star, and Early Bigi formed a fourth cluster. The PCA model included six components and showed good descriptive and predictive performance (*R* ^2^ *X*(cum) = 0.799; *Q* ^2^(cum) = 0.717), indicating that a substantial fraction of the leaf metabolomic dataset was captured.

O2PLS–DA analysis highlighted cultivar‐specific metabolite associations (Figure 7b–d). The cultivar‐based model, built using four cultivar groups as class labels, included three predictive and three orthogonal components and showed high descriptive and predictive performance (*R* ^2^ *X*(cum) = 0.784; *R* ^2^ *Y*(cum) = 0.939; *Q* ^2^(cum) = 0.932), supporting the presence of a reproducible cultivar‐associated component in leaf metabolomic profiles. Sandra leaves were characterized by elevated levels of hydroxycinnamic acids, including caffeoyl threonic acid derivatives and neochlorogenic acid, as well as phloridzin and benzyl alcohol hexose hexose. Durone Rosso and Ferrovia showed higher abundance of glycosylated and acylated coumaroyl derivatives, caffeoyl hexose, caffeoyl threonic acid deoxyhexoside, feruloyl quinic acid isoform 2, amygdalin, and the malonylated and acetylated forms of kaempferol‐O‐hexoside. Romana was associated with caffeoyl coumaroyl threonic acid and coumaroyl threonic acid. Burlat, Grace Star, and Early Bigi were characterized by higher levels of dicaffeoyl quinic acid isomers and quercetin glycosides.

Similarly to fruits, the leaf PCA score plot was also represented by coloring samples according to growing season and orchard of origin (Figure S2a,d). Cultivar‐associated grouping remained more evident than year‐ or orchard‐associated separation in the unsupervised analysis. Because the two growing seasons differed in meteorological conditions, a supervised OPLS–DA model was further built to evaluate the year effect in leaf samples (Figure S2b,c). This model separated samples according to growing season and showed good predictive ability (*R* ^2^ *X*(cum) = 0.775; *R* ^2^ *Y*(cum) = 0.856; *Q* ^2^(cum) = 0.774), suggesting that inter‐annual environmental variation contributed to the leaf metabolome. However, the year effect was not clearly visible in the unsupervised PCA, and the predictive component accounted for only a small fraction of the *X* variance (*R* ^2^ *X*[1] = 0.0227). Moreover, the corresponding S‐plot did not reveal metabolites strongly correlated with a specific growing season. These observations suggest that the year effect was detectable at the multivariate level but was broadly distributed across the metabolome rather than driven by strongly year‐specific individual metabolites.

The possible contribution of orchard location was also evaluated by O2PLS–DA using the nine orchards as class labels (Figure S2e,f). The model included eight predictive and two orthogonal components and explained a large fraction of the *X* variance (*R* ^2^ *X*(cum) = 0.874), but a lower fraction of the *Y* variance (*R* ^2^ *Y*(cum) = 0.416), with limited predictive ability (*Q* ^2^(cum) = 0.286). Consistently, the score plot did not show a clear orchard‐specific clustering, and the corresponding loading plot did not reveal strong orchard‐specific metabolite associations. These results suggest that orchard‐related variation was detectable in leaf samples, but its association with the metabolomic profiles was limited and not uniformly supported across orchard classes.

Overall, the different cultivar‐associated clustering patterns observed in fruits and leaves suggest that genotype‐associated regulation of metabolite biosynthesis and/or accumulation is at least partly organ‐specific. At the same time, the additional year‐ and orchard‐based models indicate that environmental variation contributed to both fruit and leaf metabolomes, particularly at the year level. Within the limits of the present experimental design, cultivar‐associated structure appeared more evident than orchard‐associated variation, while inter‐annual variation represented a detectable additional component.

### 3.6 Cultivar‐Associated Metabolites Are Conserved Across Organs

**FIGURE 8:**
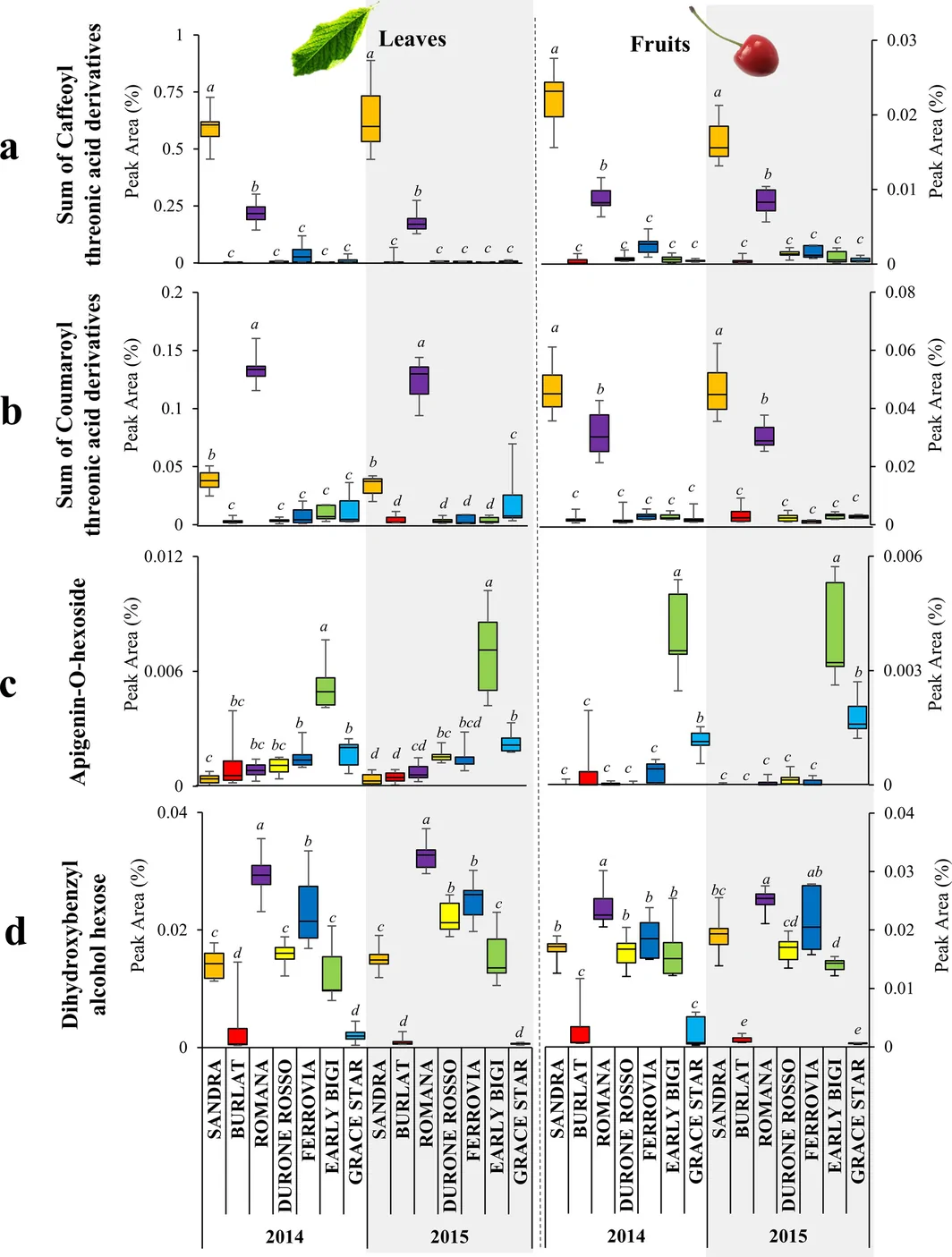
Box plots of metabolites associated with specific sweet cherry cultivars and conserved across organs and growing seasons. Data represent metabolite abundance in fruits and leaves across cultivars. Statistical analysis was performed using one‐way ANOVA followed by Tukeys HSD test (*p* < 0.05). Different letters indicate statistically significant differences among cultivars.

Overall, leaf and fruit metabolomes did not show similar cultivar‐dependent distribution patterns across the seven *Prunus avium* cultivars. However, within‐cultivar comparisons identified a subset of metabolites displaying conserved accumulation patterns in both leaves and fruits across cultivars, suggesting a shared genotype‐ (cultivar‐) dependent regulation independent of organ and growing season (Figure 8). The OPLS‐DA analysis revealed that Sandra and Romana accumulated both caffeoyl threonic acid derivatives and coumaroyl threonic acid derivatives in both fruits and leaves, with higher levels observed in fruits for the former and in leaves for the latter (Figure 8a,b). Early Bigi was specifically associated with the flavone apigenin‐O‐hexoside both in fruits and leaves (Figure 8c), while Burlat and Grace Star showed lower levels of dihydroxybenzyl alcohol hexose (Figure 8d).

## 4 Discussion

### 4.1 Genotype‐Associated Metabolomic Variation in *P. avium* Leaves and Fruits

To evaluate the relative contribution of genotype and environment to metabolite variation in cherry fruits and leaves, we designed a sampling strategy in which fruits and leaves were collected from seven distinct cultivars at a phenological stage corresponding to full fruit maturity. For each cultivar, fruit and leaf samples were obtained from trees growing in orchards located in different geographic areas and sampled across two consecutive growing seasons, thereby increasing the variability of environmental conditions. Sampling followed the strategy described by Commisso et al. (2017), with the additional collection of leaves from the same trees used for fruit sampling. The meteorological data confirmed that the two growing seasons differed, particularly in rainfall regime, number of rainy days, and solar radiation. Within this environmental context, the PCA visualizations indicated that cultivar‐associated clustering was more evident than year‐ or orchard‐associated separation, supporting the presence of a strong genotype‐associated component in fruit metabolomic variation. This observation is consistent with previous reports on sweet cherry fruit metabolomics (Picariello et al. 2016; Commisso et al. 2017; Martini et al. 2017; Di Matteo et al. 2017; Berni et al. 2021). Picariello et al. (2016) compared three sweet cherry cultivars and two sour cherry cultivars, reporting that phenolic composition, particularly anthocyanin profiles, was strongly genotype‐dependent. In our earlier investigation (Commisso et al. 2017), untargeted metabolomic profiling of fruits from 18 sweet cherry cultivars revealed cultivar‐specific associations with distinct classes of phenolic compounds, including hydroxybenzoic and hydroxycinnamic acid derivatives, anthocyanins, flavonoids, proanthocyanidins, and flavan‐3‐ols. Similarly, Martini et al. (2017) found that hydroxycinnamic acids were the most abundant metabolites in fruits of six cultivars, except for “Lapins” and “Durone della Marca”, which accumulated higher levels of anthocyanins and flavan‐3‐ols. Di Matteo et al. (2017) also reported significant variation in total flavonoid content among 26 sweet cherry cultivars grown in southern Italy between 2013 and 2015, while Berni et al. (2021) showed that flavanols and proanthocyanidins were more abundant in two local Tuscan varieties (“Morellona” and “Crognola”) than in the commercial cultivar “Durone” across three growing seasons.

A comparable genotype‐associated component was also detected in leaves. Although leaves are generally regarded as metabolically plastic and responsive to environmental variability, the additional PCA visualizations showed that cultivar‐associated patterns remained detectable across the two growing seasons and orchard locations. To the best of our knowledge, the number of studies exploring the metabolic profile of sweet cherry leaves in relation to genotype is extremely limited. Most previous investigations have focused on the effects of environmental conditions, rootstock, or agricultural practices using one or two cultivars only (Vosnjak et al. 2021; Gerasko et al. 2022; Serapicos et al. 2022; Kubes et al. 2024; Zhang et al. 2024), or have evaluated potential health‐promoting properties of leaf extracts (Jesus et al. 2019), thus preventing a comprehensive assessment of genotype‐metabolome relationships. Studies including more than two cultivars are relatively scarce and have generally aimed at exploiting leaves, considered a by‐product, as natural sources of phenolic compounds. For instance, sweet cherry leaves from “Burlat”, “Kordia”, and “Regina” collected during 2015–2016 (Dziadek et al. 2018) showed cultivar‐ and season‐dependent differences in total polyphenol content. Likewise, leaves from “Kordia”, “Regina”, “Vega”, “Hedelfińska”, “Vanda”, and “Summit” collected in 2016 (Dziadek et al. 2019) displayed genotype‐related variations in vitamin C content, antioxidant capacity, total phenolics, and selected metabolites such as chlorogenic acid, p‐coumaric acid, ferulic acid, and myricetin. Among these metabolites, chlorogenic acid and p‐coumaric acid were more characteristic of the Kordia cultivar, whereas ferulic acid and myricetin were more abundant in Regina than in the other cultivars (Dziadek et al. 2019).

Overall, our findings indicate that genetic background contributes substantially to the organization of phenolic profiles in both fruits and leaves, while year‐ and orchard‐associated variation should be acknowledged as environmental components contributing to the observed metabolomic variability. The comparison between unsupervised and supervised analyses further supports this interpretation. In both organs, cultivar‐associated grouping was evident in PCA, whereas year‐associated variation was mainly resolved by supervised modeling. This indicates that inter‐annual environmental variation contributed to the metabolomic profiles, but did not override the cultivar‐associated structure observed in the exploratory analyses. Orchard‐related variation was also detectable, although it was less clearly structured and less consistently associated with metabolite profiles than cultivar‐ or year‐associated variation.

Therefore, the data do not exclude an environmental contribution to phenylpropanoid variation. Rather, they suggest that, within the environmental range and sampling design considered here, cultivar‐associated metabolic organization remained clearly detectable in both fruits and leaves. This is particularly relevant for leaves, which are generally considered more responsive to environmental conditions than fruits, and indicates that a reproducible genotype‐associated component of phenylpropanoid metabolism can also be observed in vegetative tissues.

### 4.2 Organ‐Specific Accumulation and Structural Diversity of Phenolic Compounds in *Prunus avium*

Beyond these genotype‐associated patterns, our data also revealed a marked organ‐specific specialization of phenylpropanoid metabolism between fruits and leaves, reflecting possible and distinct physiological roles.

Our results revealed that sweet cherry leaves accumulate substantially higher levels of secondary metabolites than fruits, both in terms of total abundance and structural diversity. Despite the scarcity of data in literature, the few studies investigating the content of secondary metabolites in sweet cherry leaves confirmed that leaves contain more polyphenols than fruits. In detail, leaves of cultivars “Burlat”, “Kordia”, and “Regina”, collected during the 2015 and 2016 growing seasons at the Experimental Station of the Department of Pomology and Apiculture, University of Agriculture in Kraków (Poland), contained markedly higher concentrations of total polyphenols than the corresponding fruits, reaching 5077–12,844 mg per 100 g DW in leaves and 1988–3063 mg per 100 g DW in fruits (Dziadek et al. 2018). In a subsequent study (Dziadek et al. 2019), leaves and fruits collected in 2016 from additional cultivars, such as “Kordia”, “Regina”, “Vega”, “Hedelfińska”, and “Vanda” from Sandomierz, and “Kordia”, “Regina”, and “Summit” from Szczodrkowice, showed the same trend, with leaves containing 6012–15,318 mg per 100 g DW of total polyphenols, while fruits ranged from 1604 to 4045 mg per 100 g DW. In both reports, the difference between leaves and fruits was consistent across cultivars and sites, and, in the former study, across growing seasons, confirming that leaves represent a richer source of phenolic compounds.

Beyond sweet cherry, similar organ‐specific differences have been documented in other fruit‐bearing species. Teleszko and Wojdyło (2015) showed that leaves of *Malus domestica* , *Cydonia oblonga* , *Vaccinium macrocarpon* , and *Vaccinium myrtillus* contained significantly higher concentrations of polyphenols than fruits. Comparable trends have been reported for *Schisandra chinensis* (Mocan et al. 2014), reinforcing the view that vegetative tissues, particularly leaves, act as major reservoirs of polyphenolic metabolites.

Within the phenolic fraction, LC–MS profiling revealed that sweet cherry leaves were dominated by hydroxycinnamic acids and flavonols, together accounting for more than half of the total ion signal. In contrast, and in line with previously published data (Martini et al. 2017), anthocyanins, along with hydroxycinnamic acids and flavan‐3‐ols, predominated in mature fruits, representing nearly half of the detected compounds.

Regarding hydroxycinnamic acids, fruits mainly accumulated simpler forms, particularly neochlorogenic acid (5‐O‐caffeoylquinic acid; 7–37 mg per 100 g FW), p‐coumaroylquinic acid (5–19 mg per 100 g FW), and chlorogenic acid (3‐O‐caffeoylquinic acid; 3–4 mg per 100 g FW), in agreement with previous studies (Antognoni et al. 2020; González‐Gómez et al. 2010). Leaves, in contrast, accumulated both simple and more complex hydroxycinnamic acids at much higher concentrations, particularly chlorogenic acid (203–343 mg per 100 g FW), neochlorogenic acid (49–189 mg per 100 g FW), and p‐coumaroylquinic acid (8–82 mg per 100 g FW). Additionally, our LC–MS data revealed the presence of hydroxycinnamic acids conjugated with a compound deriving from the breakdown of L‐ascorbic acid, that is, threonic acid (Ford et al. 2024). These metabolites have already been reported in the moss *Physcomitrella patens* (Renault et al. 2017) and in leaves of *Fagus sylvatica* L. (Cadahía et al. 2015) and, at the best of our knowledge, this is the first report of their presence in sweet cherry. More complex derivatives, especially dicaffeoylquinic acid isomers, were also detected in leaves, showing high values. These findings are consistent with previous reports describing the predominance of caffeoylquinic acid derivatives in cherry foliar tissues, although published concentrations are often expressed on a dry‐weight basis or derived from concentrated extracts. For example, Nunes et al. (2021) identified a complex mixture of caffeoylquinic and dicaffeoylquinic isomers as the dominant hydroxycinnamic acids in sweet cherry cv. “Saco” leaves. In detail, the total amount of extracted hydroxycinnamic acids was declared to be 51,345.69 μg g^−1^ DW in infusions and up to 57,605.22 μg g^−1^ DW in hydroethanolic extracts, with trans‐5‐caffeoylquinic acid (neochlorogenic acid) as the major compound, followed by chlorogenic acid (3‐O‐caffeoylquinic acid). Likewise, Jesus et al. (2019) reported that hydroxycinnamic acids represent more than 70% and 60% of the total phenolic compounds in the infusion and hydroalcoholic extracts of sweet cherry leaves, respectively.

Hydroxycinnamic acids such as neochlorogenic, chlorogenic, p‐coumaroylquinic, dicaffeoylquinic, and coumaroyl‐caffeoylquinic acids have been suggested to play roles in response towards abiotic stresses, including drought, heavy metal, and temperature stresses, and towards biotic stresses against pathogen attacks by strengthening plant cell walls and acting as antimicrobial agents (Khawula et al. 2023). In particular, hydroxycinnamic acids appear to participate in regulating cell wall plasticity, either by modulating reactive oxygen species (ROS) levels or by serving as precursors of monolignols, thereby influencing the lignification process (Khawula et al. 2023).

Sweet cherry leaves also accumulated high levels of flavonoids, particularly flavonols such as quercetin‐3‐O‐rutinoside and kaempferol‐3‐O‐rutinoside, with concentrations ranging from 70 to 119 mg per 100 g FW and 111 to 161 mg per 100 g FW, respectively. Although not directly comparable due to the use of lyophilized material in other studies, similar trends were reported for sweet cherry cv. “Saco” leaves (Nunes et al. 2021; Jesus et al. 2019). In those works, quercetin‐3‐O‐rutinoside reached 673 mg per 100 g DW (Jesus et al. 2019) and 365 mg per 100 g DW (Nunes et al. 2021) in hydroethanolic leaf extracts, respectively. Kaempferol‐3‐O‐rutinoside reached 313 mg per 100 g DW in hydroethanolic leaf extracts (Jesus et al. 2019) and 130 mg per 100 g DW in extracts after infusion procedure (Nunes et al. 2021). Flavonols, particularly quercetin derivatives, act as effective antioxidants and also modulate key processes related to plant development and stress responses. Their accumulation is enhanced under abiotic stress, and these compounds contribute to the protection of photosynthetic tissues by scavenging singlet oxygen, preserving chloroplast envelope integrity, and inhibiting ROS formation (Agati et al. 2012).

Mature fruits also accumulated other phenolic compounds, particularly anthocyanins and proanthocyanidins. Cyanidin‐3‐O‐glucoside and cyanidin‐3‐O‐rutinoside were the predominant anthocyanins in fruits, with concentrations consistent with previous reports (Usenik et al. 2008; González‐Gómez et al. 2010; Antognoni et al. 2020; Martini et al. 2017). These pigments contribute to fruit coloration and antioxidant protection during ripening, while proanthocyanidins may enhance astringency and participate in defence mechanisms (Mozetič et al. 2004; Clodoveo et al. 2023; Campbell et al. 2024; Zhao et al. 2024).

Overall, the marked enrichment of hydroxycinnamic acids and flavonols in leaves, together with the dominance of anthocyanins and flavan‐3‐ols in fruits, highlights the organ‐specific specialization of phenolic metabolism in sweet cherry, reflecting distinct physiological roles in photoprotection, defence, and fruit quality.

Within this framework, our results suggest that phenylpropanoid metabolism in sweet cherry is organized into tissue‐specific metabolic channels whose structure includes a substantial genotype‐associated component. Importantly, the detection of a reproducible, genotype‐dependent organization also in leaves, a vegetative tissue traditionally regarded as metabolically plastic, indicates that genetic background contributes to phenylpropanoid pathway organization across organs, together with organ identity and environmental context.

## 5 Conclusion

This study provides a comprehensive, multi‐organ and multi‐cultivar metabolomic characterization of *Prunus avium* , highlighting the interplay between genotype, organ specificity, and environmental variability in shaping phenylpropanoid metabolism.

By integrating untargeted and quantitative metabolomic analyses of leaves and fruits collected across different orchards and growing seasons, our results indicate that cultivar‐associated variation represents a major component of specialized metabolite accumulation in both organs, within the environmental range considered here. At the same time, a pronounced organ‐specific specialization of phenylpropanoid metabolism was observed. Fruit and leaf metabolomes displayed largely independent cultivar‐dependent accumulation patterns, suggesting that genotype‐related regulation of secondary metabolism operates in an organ‐specific manner. This finding is consistent with the distinct physiological functions of vegetative and reproductive tissues, in which similar classes of metabolites may fulfill different roles or have different biological significance.

Notably, despite this overall independence, a subset of low‐abundance metabolites exhibited conserved cultivar‐dependent accumulation patterns in both leaves and fruits. These compounds may reflect shared regulatory mechanisms and potentially similar functional relevance across organs, pointing to a coordinated component of phenylpropanoid metabolism that transcends tissue specialization.

Collectively, these findings expand current knowledge of specialized metabolism in sweet cherry by showing that, within each organ, phenolic profiles contain a reproducible genotype‐associated component that remains detectable across different growing seasons and orchard locations. By incorporating leaf metabolomics alongside fruit profiling, this work underscores the importance of multi‐organ approaches for elucidating genotype‐dependent metabolic variation and provides a valuable framework for future studies on phenylpropanoid function, plant adaptation, and the valorisation of sweet cherry genetic resources.

## Author Contributions

M.C. and F.G. conceptualized the research. M.C. performed data curation. G.A., S.G., A.B., M.B., S.C., and F.D.C. conducted formal analyses. M.C., G.A., S.G., and S.N. analyzed metabolite data. F.G. acquired the fund and managed its administration. M.C. and F.G. supervised the research. M.C., F.G., and S.N. wrote and revised the manuscript. All authors read and approved the manuscript.

## Funding

This work was supported by Regione Veneto and Veneto Agricoltura, DGR 2860.

## Disclosure

Use of generative AI and AI‐assisted technologies: During the preparation of this manuscript, the authors used generative artificial intelligence tools (ChatGPT, OpenAI), accessed through an institutional educational license provided by the University of Verona, to support English language refinement and clarity of expression. All content was personally reviewed, edited, and approved by the authors, who take full responsibility for the accuracy and integrity of the manuscript.

## Conflicts of Interest

The authors declare no conflicts of interest.

## Supporting information


**Figure S1:** Multivariate analysis of fruit metabolomic profiles according to growing season and orchard of origin. PCA score plots were visualized according to growing season (a) and orchard (d). Supervised OPLS‐DA was used to evaluate year‐associated variation, showing the score scatter plot (b) and the corresponding S‐loading plot (c). Supervised O2PLS‐DA was used to evaluate orchard‐associated variation, showing the score scatter plot (e) and the corresponding loading plot (f).

**Figure S2:** Multivariate analysis of leaf metabolomic profiles according to growing season and orchard of origin. PCA score plots were visualized according to growing season (a) and orchard (d). Supervised OPLS‐DA was used to evaluate year‐associated variation, showing the score scatter plot (b) and the corresponding S‐loading plot (c). Supervised O2PLS‐DA was used to evaluate orchard‐associated variation, showing the score scatter plot (e) and the corresponding loading plot (f).

**File S1:** Datamatrix including the list of putatively identified metabolites.
**File S2:** Heat map data showing the relative abundance of metabolites selected from the O2PLS–DA loading plots for fruits and leaves.

**Table S1:** Agronomical data.
**Table S2:** Meteorological data from the three weather stations representative of the sampled production area during the 2014 and 2015 growing seasons.
**Table S3:** Metabolite values (mg/100 g fresh weight) per growing season.
**Table S4:** Metabolite values (mg/fruit or leaf) per growing season.

## Data Availability

The raw LC‐HRMS data generated in this study have been deposited in the MassIVE repository under accession number MSV000102859 (https://doi.org/10.25345/C5MKE5P65). Processed data are provided in the article and its Supporting Information.
